# Genomic analysis of *Shigella* isolates from Lebanon reveals marked genetic diversity and antimicrobial resistance

**DOI:** 10.1099/mgen.0.001157

**Published:** 2023-12-15

**Authors:** Iman Yassine, Rayane Rafei, Maria Pardos de la Gandara, Marwan Osman, Laetitia Fabre, Fouad Dabboussi, Monzer Hamze, François-Xavier Weill

**Affiliations:** ^1^​ Institut Pasteur, Université Paris Cité, Unité des Bactéries pathogènes entériques, Centre National de Référence des Escherichia coli, Shigella et Salmonella, Paris, F-75015, France; ^2^​ Laboratoire Microbiologie Santé et Environnement (LMSE), Doctoral School of Sciences and Technology, Faculty of Public Health, Lebanese University, Tripoli, Lebanon; ^3^​ Department of Neurosurgery, Yale University School of Medicine, New Haven, CT 06510, USA; ^†^​ Present address: Nuffield Department of Population Health, University of Oxford, Oxford OX3 7LF, UK

**Keywords:** antibiotics, Gram-negative bacteria, resistance, whole genome sequencing, *Shigella*, serotyping

## Abstract

In this study, we characterized 54 clinical isolates of *Shigella* collected in North Lebanon between 2009 and 2017 through phenotypic and genomic analyses. The most prevalent serogroup was *S. sonnei,* accounting for 46.3 % (25/54) of the isolates, followed by *S. flexneri* (27.8 %, 15/54), *S. boydii* (18.5 %, 10/54) and *S. dysenteriae* (7.4 %, 4/54). Only three isolates were pan-susceptible, and 87 % (47/54) of the isolates had multidrug resistance phenotypes. Notably, 27.8 % (15/54) of the isolates were resistant to third-generation cephalosporins (3GCs) and 77.8 % (42/54) were resistant to nalidixic acid. 3GC resistance was mediated by the extended-spectrum beta-lactamase genes *bla*_CTX-M-15_ and *bla*_CTX-M-3_, which were present on various plasmids. Quinolone resistance was conferred by single point mutations in the *gyrA* DNA gyrase gene, leading to GyrA S83L, GyrA D87Y or GyrA S83A amino acid substitutions. This is the first study, to our knowledge, to provide genomic insights into the serotypes of *Shigella* circulating in Lebanon and the various antimicrobial resistance determinants carried by these strains.

## Data Summary

The authors confirm that all the supporting data are provided within the article or in the supplementary data files. The short-read sequence data generated in this study have been submitted to EnteroBase (https://enterobase.warwick.ac.uk/) and to the European Nucleotide Archive (ENA, https://www.ebi.ac.uk/ena/) under study number PRJEB63380. All the accession numbers of the short-read sequences are listed in Data S1, available in the online version of this article. The plasmid sequences obtained have been deposited in GenBank (https://www.ncbi.nlm.nih.gov/genbank/) under accession numbers OR237793–OR237802 (Table 2).

**Table 1.** Genomic characteristics and antibiotic resistance profiles of the *Shigella* isolates analysed in this study

Serogroup	Genotype (*N*)	AMR (*N*)	Acquired AMR genes	Mutation in QRDR
* S. sonnei *	3.6.1 (20)	STR SUL TMP TET NAL (11)	*strA, strB, sul2, dfrA1, tet(A)*	*gyrA* (S83L)
		AMP CTX STR GEN SUL TMP TET NAL (4)	*bla*_CTX-M-15_ *, bla*_TEM-1B_ *, strA, strB, aac(3)-IId, sul2, dfrA1, tet(A*)	*gyrA* (S83L)
		AMP CTX STR SUL TMP TET NAL (3)	*bla*_CTX-M-3_ *, strA, strB, sul2, dfrA1, tet(A)*	*gyrA* (S83L)
		AMP CTX CAZ GEN TMP NAL (1)	*bla*_CTX-M-15_ *, bla*_TEM-1B_ *, aac(3)-IId, dfrA1*	*gyrA* (S83L)
		TMP NAL (1)	*dfrA1*	*gyrA* (S83L)
	3.6.3 (5)	STR SUL TMP TET NAL (2)	*strA, strB, sul2, dfrA1, tet(A)*	*gyrA* (D87Y)
		TMP NAL (2)	*dfrA1*	*gyrA* (D87Y)
		NAL (1)	–	*gyrA* (D87Y)
* S. flexneri * 1–5, X, Y	PG1a (3)	AMP STR CHL TET (1)	*bla*_OXA-1_ *, aadA1, catA1, tet(B)*	–
		AMP STR TMP CHL TET (1)	*bla*_OXA-1_ *, aadA1, dfrA1, catA1, tet(B)*	–
		AMP CTX CAZ STR GEN TMP TET (1)	*bla*_CTX-M-15_ *, bla*_TEM-1B_ *, aadA1, aac(3)-IId, dfrA1, tet(B)*	–
	PG3 (3)	STR TMP TET NAL (1)	*aadA1, dfrA1, tet(B)*	*gyrA* (S83L)
		AMP CTX STR SUL TMP CHL TET (1)	*bla*_CTX-M-3_ *, bla*_OXA-1_ *, strA, strB, aad1, sul2, dfrA1, catA1, tet(B)*	–
		Susceptible (1)	–	–
* S. flexneri* 6	S1b (9)	STR SUL TMP TET NAL (7)	*aadA1, sul2, dfrA1, tet(B)*	*gyrA* (D87Y)
		AMP CTX STR SUL TMP NAL (1)	*bla*_CTX-M-3_ *, aad1, sul2, dfrA1*	*gyrA* (D87Y)
		AMP CTX CAZ STR TMP NAL (1)	*bla*_CTX-M-15_ *, aad1, dfrA1*	*gyrA* (D87Y)
* S. boydii *	S1c (8)	STR TMP NAL (5)	*aadA1, dfrA1*	*gyrA* (S83L)
		AMP CTX CAZ STR SUL TMP (1)	*bla*_CTX-M-15_ *, bla*_TEM-1B_ *, strA, strB, sul2, dfrA14, qnrS1*	–
		AMP CTX CAZ STR SUL TMP NAL CIP (1)	*bla*_CTX-M-15_ *, aadA1, sul1, sul2, dfrA1, dfrA5, tet(A), qnrS1*	*gyrA* (S83L)
		Susceptible (1)	–	–
	S1b (2)	AMP CTX CAZ GEN (1)	*bla*_CTX-M-15_ *, bla*_TEM-1B_ *, aac(3)-IId*	–
		Susceptible (1)	–	–
* S. dysenteriae *	S1a (3)	AMP STR CHL TET (2)	*bla*_OXA-1_ *, aadA1, catA1, tet(B)*	–
		AMP STR SUL TMP CHL TET NAL (1)	*bla*_OXA-1_ *, strA, strB, aadA1, sul2, dfrA1, catA1, tet(B)*	*gyrA* (S83A)
	S2d (1)	AMP STR SUL TMP (1)	*bla*_TEM-1B_ *, strA, strB, sul2, dfrA14*	–

AMP, ampicillin; AMR, antimicrobial resistance; CAZ, ceftazidime; CHL, chloramphenicol; CIP, ciprofloxacin; CTX, cefotaxime; GEN, gentamicin; NAL, nalidixic acid; QRDR, quinolone resistance-determining region; STR, streptomycin; SUL, sulfonamides; TET, tetracycline; TMP, trimethoprim.

**Impact Statement**

Shigellosis poses a significant threat to public health, particularly in low- and middle-income countries, where it predominantly affects young children. The severity of the illness, coupled with the ability of *Shigella* to acquire multiple antimicrobial resistance determinants, further exacerbates the problem. Effective surveillance of shigellosis is recognized as crucial for informed decision-making and appropriate public health interventions. However, the epidemiology of *Shigella* remains poorly understood in Lebanon, a country facing unique challenges of economic instability and poor healthcare infrastructure. We addressed this gap in our knowledge by characterizing 54 *Shigella* isolates from North Lebanon, focusing on their prevalence, antimicrobial susceptibility profiles and genotypic characteristics. Our findings reveal considerable diversity among the circulating *Shigella* strains, with a high proportion displaying antimicrobial drug resistance. Resistance to third-generation cephalosporins was mediated by *bla*_CTX-M-15_ and *bla*_CTX-M-3_ genes carried on IncI1 and IncFIB plasmids. Our findings highlight the critical need for ongoing surveillance efforts to provide an accurate assessment of the burden of disease due to shigellosis and to provide essential guidance for its management. The whole-genome sequencing data presented here will also serve as a valuable resource for future research investigating the evolutionary patterns of antimicrobial susceptibility in Lebanon, thereby deepening our understanding of the mechanisms and dynamics underlying the spread of *Shigella* strains in the region.

## Introduction

*Shigella *, a Gram-negative bacterium from the family *Enterobacteriaceae *, is a major cause of diarrhoeal disease, accounting for about 210 000 deaths annually [1, 2]. *Shigella* is mostly transmitted through contaminated food, water or person-to-person contact. It can cause mild diarrhoea, severe dysentery with bloody stools and potentially fatal dehydration, especially in vulnerable populations, such as young children, the elderly and immunocompromised individuals [3, 4].

The genus *Shigella* encompasses four serogroups: *S. dysenteriae, S. boydii*, *S. flexneri* and *S. sonnei *. Cases of shigellosis in low- and middle-income countries (LMICs), where the disease burden is highest, are mostly caused by the last two of these serogroups [1, 4–6]. Despite the public health impact of shigellosis, there is currently no licensed vaccine against *Shigella *, primarily due to its substantial genomic and phenotypic diversity, which poses a major challenge in vaccine development [7, 8]. Consequently, the management of shigellosis relies on supportive care and antimicrobial therapy. However, the increasing frequency of antimicrobial resistance (AMR) in *Shigella* strains has become a major concern, leading the World Health Organisation (WHO) to classify *Shigella* as a priority pathogen for which new antimicrobial drugs are urgently required [2, 9–11].

According to global disease burden estimates for 2016, the incidence of episodes of diarrhoea attributable to *Shigella* among children under the age of 5 years was 107.3 episodes per 1000 child-years in the Middle East and North Africa [1]. In Lebanon, the national burden of *Shigella* infections remains unclear as the dysentery cases reported by the Ministry of Public Health (https://www.moph.gov.lb/en/Pages/2/194/surveillance-data) group together the infections caused by *Entamoeba histolytica* and those caused by *Shigella* spp. This lack of clarity is compounded by the limited epidemiological data for *Shigella* in Lebanon due to the lack of robust and sustainable epidemiological surveillance programmes and the challenges faced by most clinical laboratories in the identification of *Shigella* and its differentiation from enteroinvasive *Escherichia coli* (EIEC) [12, 13]. A study recently conducted in two Lebanese tertiary healthcare settings showed that EIEC and *Shigella* accounted for 18.9 % of the enteric pathogens isolated from patients with acute community-acquired diarrhoea [12]. Lebanon is currently grappling with calamitous challenges to its infrastructure and political stability, together with pollution, associated with a severe economic collapse that resulted in an acute devaluation of its currency by more than 95 % and shortages of imported goods, limiting the availability and increasing the prices of essential goods, with an impact on public health, including critically important antimicrobial drugs (CIAs) [14]. Furthermore, the country is currently providing shelter for about 1.5 million refugees displaced by the ongoing conflict in Syria [15]. In this context, an understanding of the distribution of *Shigella* serotypes and associated antimicrobial susceptibility patterns in Lebanon is crucial for epidemiological surveillance and vaccine development, which is also a WHO priority [7]. We therefore performed phenotypic and in-depth genomic analyses of *Shigella* isolates collected in North Lebanon to provide baseline information on the distribution of *Shigella* serotypes and AMR determinants in the country.

## Methods

### *Shigella* isolates

Fifty-four *Shigella* spp. isolates were obtained from clinical stool samples of patients admitted to tertiary healthcare facilities for bacillary dysentery between 2009 and 2017 [Nini Hospital (*n*=37), Al-Haykal Hospital (*n*=3), Dar Al-Chifae Hospital (*n*=4), El Youssef Hospital Centre (*n*=4) and Tripoli Governmental Hospital (*n*=6)]. The isolates from Nini Hospital identified as *Shigella* spp. were collected throughout the study period, whereas those from El Youssef Hospital were collected from April 2015. Isolates were collected on a random basis from the other hospitals. Isolates were initially identified with API 20E strips (bioMérieux) or with the RapID ONE system (Remel). They were then transported to the Laboratoire Microbiologie Santé et Environnement (LMSE) in Tripoli, Lebanon, where they were stored in the Collection Microbiologique de l’Université Libanaise (CMUL). For characterization and further analyses, the isolates were subsequently shipped to the French National Reference Centre for *Escherichia coli *, *Shigella* and *Salmonella* (FNRC-ESS) at the Institut Pasteur, Paris, France, in accordance with international regulations.

### *Shigella* identification and serotyping

*Shigella* typing was performed at the FNRC-ESS. Cultures were grown overnight at 37 °C on Drigalski agar. The isolates were first subjected to biochemical tests for lactose, motility, lysin decarboxylase (LDC), hydrogen sulphide (H_2_S), mucate, tetrathionate reductase, glycerol, glucose (acid and gas), d-mannitol, indole, ornithine decarboxylase (ODC), ortho-nitrophenyl-β-d-galactopyranoside (ONPG), dulcitol, rhamnose and d-xylose. Serotyping was performed with slide agglutination assays using commercially available polyvalent and monovalent somatic (O)-grouping antisera (Denka Seiken; Sifin Diagnostics) and in-house antisera from the FNRC-ESS.

### Antimicrobial susceptibility testing

Antimicrobial susceptibility testing was performed by the disc diffusion method on Mueller-Hinton (MH) agar for the following antimicrobial drugs: ampicillin (AMP, 10 µg), cefotaxime (CTX, 5 µg), ceftazidime (CAZ, 10 µg), ertapenem (ERT, 10 µg), streptomycin (STR, 10 µg), gentamicin (GEN, 10 µg), amikacin (AKN, 30 µg), tigecycline (TGC, 15 µg), sulfonamides (SUL, 200 µg), trimethoprim (TMP, 5 µg), chloramphenicol (CHL, 30 µg), tetracycline (TET, 30 µg), nalidixic acid (NAL, 30 µg), ciprofloxacin (CIP, 5 µg), pefloxacin (PEF, 5 µg) and azithromycin (AZM, 15 µg). The results were interpreted according to the 2020 guidelines of the antibiogram committee of the French Society for Microbiology (CA-SFM)/European Committee on Antimicrobial Susceptibility Testing (EUCAST) (https://www.sfm-microbiologie.org/casfm/). Minimum inhibitory concentrations (MICs) for NAL, CIP and AZM were determined with E-test strips (Biodisk; bioMérieux) for all isolates. The MICs of ceftriaxone (CRO), CAZ and imipenem (IMP) were determined with E-test strips for those isolates found to be resistant to CTX or CAZ by the disc diffusion method. Resistance to third-generation cephalosporins (3GCs) was defined as resistance to CAZ, CTX or CRO. The Clinical and Laboratory Standards Institute (CLSI) criteria were then used for final interpretation [16]. We differentiated *Shigella* isolates that were wild-type (WT) and susceptible to CIP from those that were non-WT, by defining two categories based on the epidemiological cutoffs used by the CLSI for *Salmonella* spp.: decreased susceptibility to CIP (MIC>0.06 mg l^−1^ and ≤0.5 mg l^−1^) and true susceptibility to CIP (MIC≤0.06 mg l^−1^) [16].

### Whole-genome sequencing and processing

Total DNA was extracted with the MagNA Pure DNA isolation kit (Roche Molecular Systems) from overnight cultures in tryptic soy broth (TSB) at 37 °C. Whole-genome sequencing (WGS) was performed at the genomics platform of the Institut Pasteur, in Paris, France (Plateforme de microbiologie mutualisée, P2M). Libraries were constructed with the Nextera XT kit (Illumina) and sequencing was performed with the NextSeq 500 system (Illumina), generating 150 bp paired-end reads. The short reads were filtered with FqCleanER version 21.06 (https://gitlab.pasteur.fr/GIPhy/fqCleanER), and assembled *de novo* with SPAdes version 3.15 [17]. The short reads were also uploaded onto EnteroBase (https://enterobase.warwick.ac.uk) and passed its quality control criteria (sequence length: 3.7–6.4 Mb, number of contigs: ≤800, N_50_: >20 kb, proportion of N’s: <3 %, species assignment according to Kraken: >70 % contigs assigned) [18]. Between 98.4 and 100 % of the contigs from the 54 genomes were assigned to *Escherichia coli */* Shigella *.

Five extended-spectrum beta-lactamase (ESBL)-producing *Shigella* isolates were selected and sequenced with a Nanopore MinION sequencer (Oxford Nanopore Technologies) as previously described [9]. Briefly, DNA was extracted from the isolates grown overnight in alkaline nutrient agar at 37 °C, and then cultured in brain-heart infusion (BHI) broth at 37 °C with shaking to a final OD_600_ of 0.8. The bacterial cells were then harvested, and genomic DNA was extracted with Qiagen Genomic-tip 100 G^−1^ columns, according to the manufacturer’s protocol. The extracted DNA was used to prepare a library according to the instructions of the ‘Native barcoding genomic DNA (with EXP-NBD104, EXP-NBD114, and SQK-LSK109)’ procedure provided by Oxford Nanopore Technology. Sequencing was then performed with a MinION Mk1C device. Long reads were filtered with Filtlong (version 0.2.0), 95 % of the reads being retained on the basis of a minimum length of 1000 bp (https://github.com/rrwick/Filtlong). The genome sequences were assembled with UniCycler version 0.4.8 according to a hybrid approach using both the short and long reads [19]. Prokka version 1.14.5 (https://github.com/tseemann/prokka) was used for plasmid annotation [20]. Illumina short reads were mapped to constructed plasmids with BWA version 0.7.4 [21] and SAMtools version 1.13 [22].

### Phylogenetic and genotyping analysis

ShigaPass version 1.15 was used for serotype confirmation [23]. All isolates were typed with the *Escherichia */* Shigella* core-genome multilocus sequence typing (cgMLST) scheme implemented in EnteroBase [18, 24, 25]. *S. sonnei* genotyping was performed with the hierarchical single-nucleotide variant-based genotyping scheme described by Hawkey *et al.* and implemented in Mykrobe software version 0.9.0 (https://github.com/katholt/sonneityping) [11]. A single-nucleotide polymorphism (SNP)-based RAxML phylogenetic tree was generated with the EnteroBase ‘Create SNP Project’ tool, with the CL-007 genome as a reference [18]. The tree was midpoint-rooted and visualized with Interactive Tree of Life (iTOL) version 6 [26].

### Antimicrobial resistance gene analysis

AMR genes were identified with ResFinder version 4.1 (https://cge.cbs.dtu.dk/services/ResFinder/) on SPAdes and UniCycler assemblies [27]. The plasmids were typed with PlasmidFinder version 2.1.1. (https://cge.cbs.dtu.dk/services/PlasmidFinder/) [28]. The plasmids identified were compared with the known sequences in the NCBI BLASTn nucleotide collection (nr/nt) database (https://blast.ncbi.nlm.nih.gov/Blast.cgi) [29]. Finally, plasmids were aligned and visualized with BRIG version 0.95 (http://sourceforge.net/projects/brig), using the multifasta files unless otherwise specified in the figure legends [30].

## Results and discussion

### *Shigella* serotype distribution

*Shigella* serotyping was performed by slide agglutination and *in silico* with ShigaPass. No discrepancies were observed between the results of the two methods. *S. sonnei* was the most prevalent serogroup (46.3 %, 25/54) followed by *S. flexneri* (27.8 %, 15/54), which was represented by the following serotypes: *S. flexneri* 6 (16.7 %, 9/54), *S. flexneri* 2a (5.6 %, 3/54), *S. flexneri* 1b (3.7 %, 2/54) and *S. flexneri* 1c (1.8 %, 1/54). *S. boydii* was identified for 18.5 % (10/54) of all isolates, with *S. boydii* 20 being the most common serotype (11.1 %, 6/54), followed by *S. boydii* 10 (3.7 %, 2/54), *S. boydii* 2 (1.85 %, 1/54) and *S. boydii* 4 (1.85 %, 1/54). Only four isolates (7.4 %) were identified as *S. dysenteriae* and they belonged to *S. dysenteriae* 12 (3.7 %, 2/54), *S. dysenteriae* 2 (1.85 %, 1/54) and *S. dysenteriae* 3 (1.85 %, 1/54) (Data S1).

All isolates could be classified to the previously identified *Shigella* cgMLST clusters [24]. The *S. sonnei* isolates belonged to lineage 3, particularly to subclade 3.6.1 (80 %, 20/25), known as ‘CipR parent’ (Data S1) [11]. Three of the six *S. flexneri* group 1–5, X, Y isolates belonged to phylogroup (PG) 3, the other three belonging to PG1a (Data S1) [2, 24]. These results highlight the diversity of the *Shigella* serotypes circulating in North Lebanon and demonstrate the predominance of *S. sonnei *. We also detected serotypes that had emerged more recently, such as *S. boydii* 20 and *S. flexneri* 1c. *S. boydii* 20 was first described in 1999, and it became the predominant serotype of *S. boydii* in Canada within a year [31]. The emergence of *S. flexneri* 1c has also been reported in Bangladesh, Egypt, Indonesia and Pakistan [32–34].

Our study has several limitations. The number of *Shigella* isolates collected was limited because not all the participating tertiary healthcare centres sent all the *Shigella* isolates they obtained to the LMSE. In addition, shigellosis is generally a self-limiting disease with symptoms such as watery diarrhoea, and these characteristics may have resulted in many of those infected not seeking medical attention or undergoing testing. Moreover, many clinical laboratories experience difficulties identifying *Shigella* spp. with the available testing tools. Finally, this was not a nationwide study as all the participating laboratories were located in northern Lebanon. The sample studied may not therefore be entirely representative of the true prevalence and distribution of *Shigella* strains in the population.

### Antimicrobial resistance

We evaluated the susceptibility of *Shigella* isolates to a panel of antimicrobial drugs. We found that 87 % (47/54) of isolates displayed resistance to three or more antimicrobial drug classes; these isolates were considered to be multidrug-resistant (MDR) (Table 1, Data S1). Only three isolates were pan-susceptible. The proportion of isolates displaying AMR was highest for trimethoprim (85.2 %, 46/54), followed by streptomycin (83.3 %, 45/54), nalidixic acid (77.8 %, 42/54), tetracycline (64.8 %, 35/54) and sulfonamides (61.1 %, 33/54). Lower resistance rates were noted for ampicillin (38.9 %, 21/54), cefotaxime (27.8 %, 15/54), gentamicin (13 %, 7/54), ceftazidime (11.1 %, 6/54), chloramphenicol (11.1 %, 6/54) and ciprofloxacin (1.9 %, 1/54). However, 55.6 % (30/54) of the isolates displayed decreased susceptibility to ciprofloxacin. All isolates were susceptible to ertapenem, amikacin, tigecycline and azithromycin. We identified 19 antimicrobial drug resistance profiles in total, the most frequent of which combined resistance to streptomycin, sulfonamides, trimethoprim, tetracycline and nalidixic acid (37 %, 20/54) (Table 1, Data S1). International guidelines recommend the use of fluoroquinolones (e.g. ciprofloxacin), 3GCs (such as ceftriaxone, ceftazidime and cefotaxime) and macrolides (azithromycin) for the treatment of shigellosis [35, 36]. Our results suggest that azithromycin remains effective in Lebanon, but 27.8 % of isolates were resistant to 3GCs and 1.9 % were resistant to ciprofloxacin. Our focus on infections from tertiary care centres may have resulted in the inclusion of fewer isolates from patients with mild infections. In addition, it is not possible to rule out better rates of referral by the clinical laboratories participating in this study for patients infected with isolates displaying higher levels of AMR. Our findings therefore require confirmation in a larger study, and changes in the results might be expected if extensively drug-resistant (XDR) *Shigella* isolates were introduced, as already reported in various parts of the world [9, 37, 38].

### Analysis of the AMR genes

**Fig. 1.**
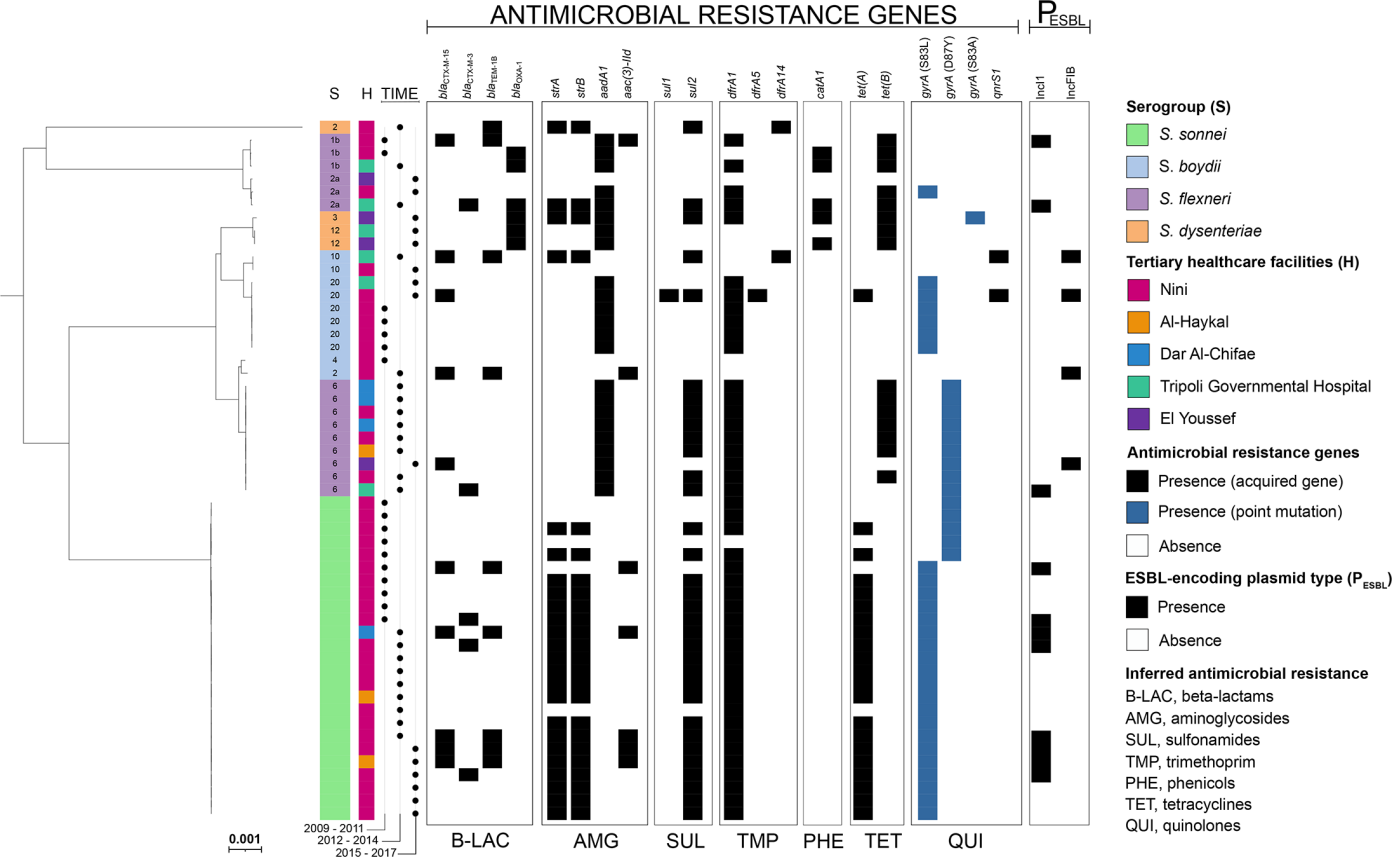
Maximum-likelihood phylogeny of 54 *Shigella* genomic sequences isolated from Lebanon. The phylogenetic tree was reconstructed with the ‘create SNP project’ tool in EnteroBase, based on the 81 516 non-repetitive SNPs present in 95 % or more of all the queried genomes. The strips show the associated information for each isolate, in the following order from left to right: (1) serogroup identity with the serotype indicated inside; (2) source of the isolate; (3) year of isolation; (4) antimicrobial resistance genes; and (5) plasmid type associated with the ESBL genes. Bar, 0.001 nucleotide substitutions per site.

In addition to performing antimicrobial susceptibility testing, we also analysed WGS data to investigate the mechanisms of resistance in more detail. Genomic analysis revealed the presence of 19 different AMR genes, including genes conferring resistance to sulfonamides (*sul1* and *sul2*), tetracycline (*tetA* and *tetB*), trimethoprim (*dfrA1, dfrA5* and *dfrA14*), and phenicols (*catA1*). Several different AMR genes were implicated in resistance to aminoglycosides. The most frequent were *strA*, *strB* and *aadA1,* found in 44.4 % (24/54), 44.4 % (24/54), and 42.6 % (23/54) of the isolates, respectively. Moreover, 46.3 % (25/54) of the isolates carried more than one aminoglycoside resistance gene (Table 1, Fig. 1, Data S1).

Quinolone resistance was present at high frequency (77.8 %, 42/54) and was mediated by a single mutation in the quinolone resistance-determining region (QRDR) of the *gyrA* gene encoding subunit A of the DNA gyrase. The GyrA S83L variant was the most frequent, found in 64.3 % (27/42) of the nalixidic acid-resistant isolates (MIC >16 mg l^−1^), followed by the GyrA D87Y variant, which was present in 33.3 % (14/42). The GyrA S83A variant was found in only one isolate. All isolates carrying the *gyrA* S83L mutation (20 *S. sonnei *, six *S. boydi* 20 and one *S. flexneri* 2a) had decreased susceptibility to ciprofloxacin (MIC >0.06 mg l^−1^ and ≤0.5 mg l^−1^). By contrast, only 21.4 % (3/14) of the isolates carrying the *gyrA* D87Y mutation displayed decreased susceptibility to ciprofloxacin, all the remaining isolates carrying this mutation being susceptible (MIC ≤0.06 mg l^−1^). This mutation was found in all nine *S. flexneri* 6 isolates and in five *S. sonnei* isolates. The *S. dysenteriae* 3 isolate with the *gyrA* S83A mutation was also susceptible to ciprofloxacin. Only two isolates had plasmid-mediated quinolone resistance (PMQR) genes. These two isolates – one *S. boydii* 10 (CL-045) and one *S. boydii* 20 (CL-056) – harboured the *qnrS1* gene. CL-056 also carried the *gyrA* S83L mutation. The combined presence of the *qnrS1* gene and the *gyrA* mutation in CL-056 led to resistance to ciprofloxacin (Table 1, Fig. 1, Data S1).

Ciprofloxacin-resistant isolates were rare in our study (a single isolate), but 55.6 % (30/54) of the *Shigella* isolates displayed decreased susceptibility to this drug, corresponding to the first step towards the development of full resistance to ciprofloxacin through the accumulation of QRDR mutations and/or PMQR genes [39]. In our study, 80 % (20/25) of the *S. sonnei* isolates – the most prevalent *Shigella* serogroup/serotype in our study – belonged to sublineage 3.6.1 (also known as ‘CipR parent’). This *S. sonnei* sublineage originated in South Asia in 2007 and has displayed a tendency to accumulate mutations in the QRDR region of the *gyrA* and *parC* genes, generating genotypes resistant to ciprofloxacin [9–11]. Caution should therefore be exercised in the use of fluoroquinolones to treat shigellosis in Lebanon.

Ampicillin resistance was conferred by the *bla*_OXA-1_ and *bla*_TEM-1B_ beta-lactamase genes, the latter of which predominated (60 %, 9/15). The *bla*_OXA-1_ gene was carried by six isolates (three *S. flexneri* and three *S. dysenteriae *), along with at least the *tet(B), aadA1* and *catA1* genes (Table 1, Fig. 1, Data S1). This combination is normally seen in the SRL pathogenicity island (PAI) conferring resistance to tetracycline, aminopenicillins, streptomycin and chloramphenicol [2, 40]. Resistance to 3GCs was conferred by two ESBL genes, *bla*_CTX-M-15_ and *bla*_CTX-M-3_, which were found in 27.7 % (15/54) of *Shigella* isolates (eight *S. sonnei *, four *S. flexneri* and three *S. boydii* isolates). The *bla*_CTX-M-15_ gene was the most common, present in 10 of the 15 isolates, the *bla*_CTX-M-3_ gene being found in the remaining five isolates (Table 1, Fig. 1, Data S1). Previous studies have also reported a high prevalence of the *bla*_CTX-M-15_ gene in *Shigella* isolates, this gene being considered the most common ESBL gene in *Shigella* [41, 42]. It is usually found in association with the *bla*_TEM-1B_ gene, and this was the case in all but two of the isolates studied here [33, 43–48]. Matar *et al.* reported the first detection of CTX-M-15-producing *Shigella* in Lebanon in 2007 [49]. The presence of both *bla*_CTX-M-15_ and *bla*_TEM-1B_ has been documented not only in *Shigella* isolates, but also in ESBL-producing *E. coli* collected from river water and from wastewater from refugee camps in Lebanon [49–52].

### Characterization of ESBL-encoding plasmids

**Fig. 2.**
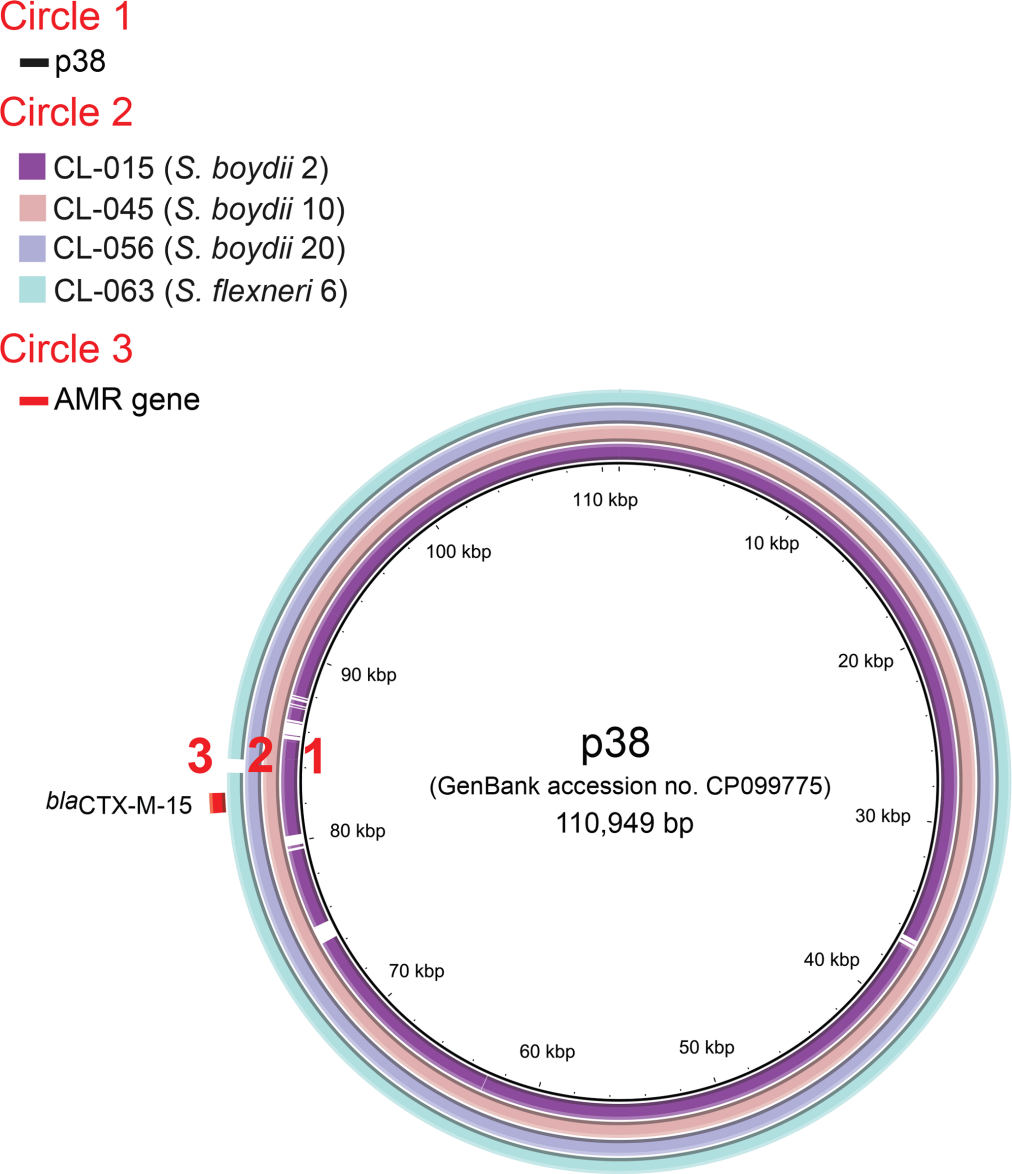
Circular map and comparative analysis of the IncFIB plasmids carrying the ESBL *bla*_CTX-M-15_ gene. Circles from inside to outside indicate: (1) the nucleotide position of p38, a plasmid from an *S. sonnei* isolate (S17BD05916) collected in Belgium in 2017 [53]; (2) regions of p38 displaying high levels of sequence identity to regions from draft genome assemblies of CL-015 (* S. boydii* 2 isolate, our study), CL-045 (* S. boydii* 10 isolate, our study), CL-056 (* S. boydii* 20 isolate, our study) and CL-063 (* S. flexneri* 6 isolate, our study); and (3) antimicrobial drug resistance (AMR) genes.

We initially investigated the location of the ESBL genes in short-read assemblies, with PlasmidFinder, Resfinder and BLASTn, by determining whether these genes were present on the same contig as the plasmid replicons. Using this approach, we found that all ESBL genes were, indeed, present on the contig carrying the plasmid replicon genes, confirming that they were plasmid-borne. Two different Inc-type plasmids were associated with the ESBL genes: IncI1 (found in 11/15 isolates resistant to 3GCs) associated with *bla*_CTX-M-15_ or *bla*_CTX-M-3_, and IncFIB (found in the remaining four isolates) associated with *bla*_CTX-M-15_ (Fig. 1, Data S1). One of the short-read assemblies (from *S. boydii* 10 isolate CL-045) revealed that the 111 026 bp contig carrying the IncFIB replicon was 99 % identical, over 100 % of the alignment, to p38 (GenBank accession no. CP099775.1), a plasmid from an *S. sonnei* isolate (S17BD05916) collected in Belgium in 2017 [53]. This alignment confirmed the presence of a complete ~111 kb IncFIB plasmid harbouring the *bla*_CTX-M-15_ gene proximal to the IS*1380* family transposase IS*Ecp1*. We investigated whether the other three isolates containing *bla*_CTX-M-15_ (CL-015, CL-056, CL-063) harboured the same IncFIB plasmid, by performing both read-mapping analysis (Table S1) and BLASTn analysis with BRIG on draft genomes against plasmid p38 (Fig. 2). There was a high degree of identity between these isolates, and all displayed >96.5 % coverage of the IncFIB plasmid, confirming the presence of very similar plasmids in each of these isolates (Fig. 2, Table S1, Data S1). This plasmid was carried by *S. boydii* isolates belonging to three different serotypes and by one *S. flexneri* 6 isolate (Figs 1 and 2, Data S1).

**Fig. 3.**
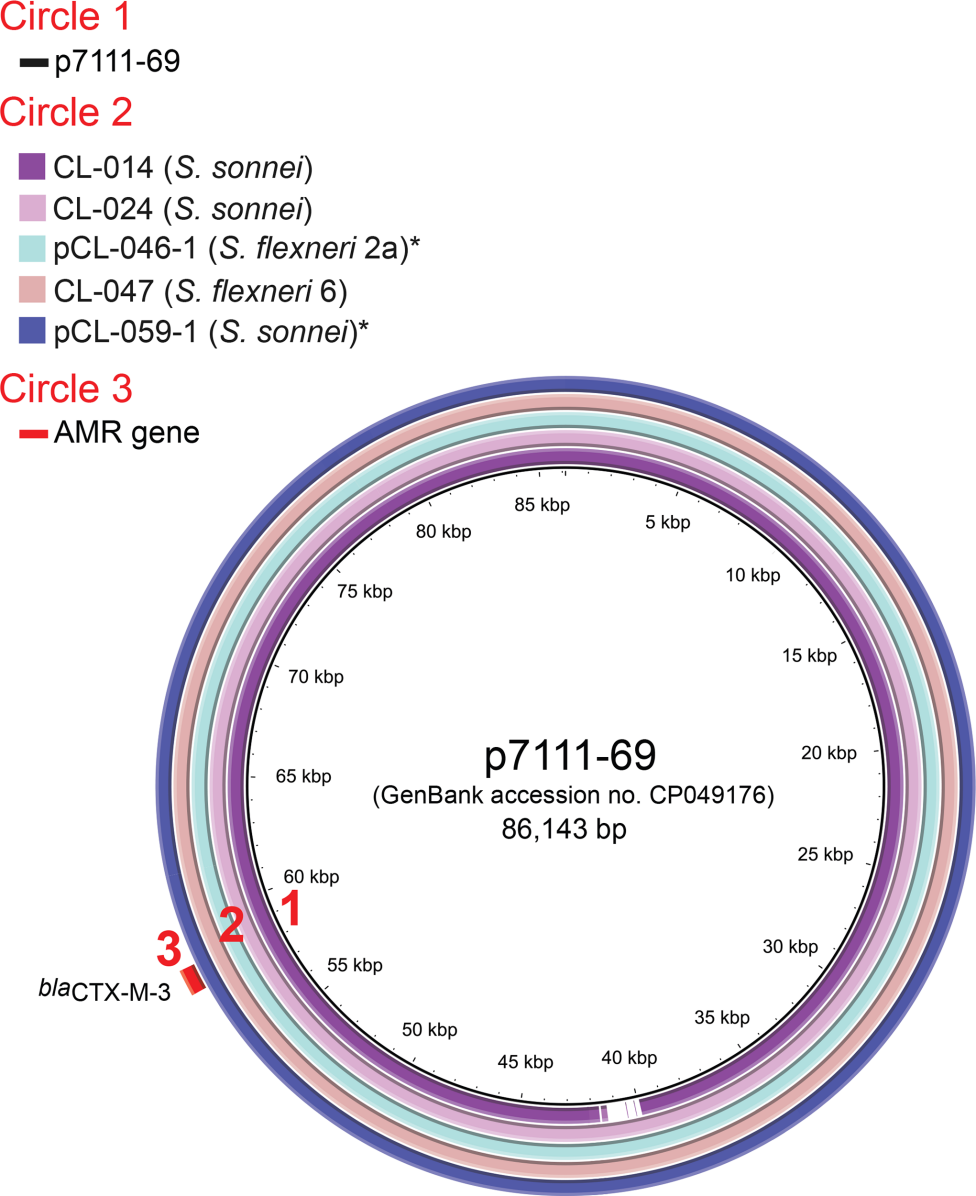
Circular map and comparative analysis of the IncI1 plasmids carrying the ESBL *bla*_CTX-M-3_ gene. Circles from inside to outside indicate: (1) the nucleotide position of p7111-69, a plasmid from *S. sonnei* isolate 7111.69 acquired in Turkey in 2019 [43]; (2) regions of p7111-69 displaying high levels of sequence identity to regions from complete or draft genome assemblies of CL-014 (* S. sonnei* isolate, our study), CL-024 (* S. sonnei* isolate, our study), CL-046 (* S. flexneri* 2a isolate, our study), CL-047 (* S. flexneri* 6 isolate, our study) and CL-059 (* S. sonnei* isolate, our study); and (3) antimicrobial drug resistance (AMR) genes. Asterisks indicate isolates with complete genomes.

**Fig. 4.**
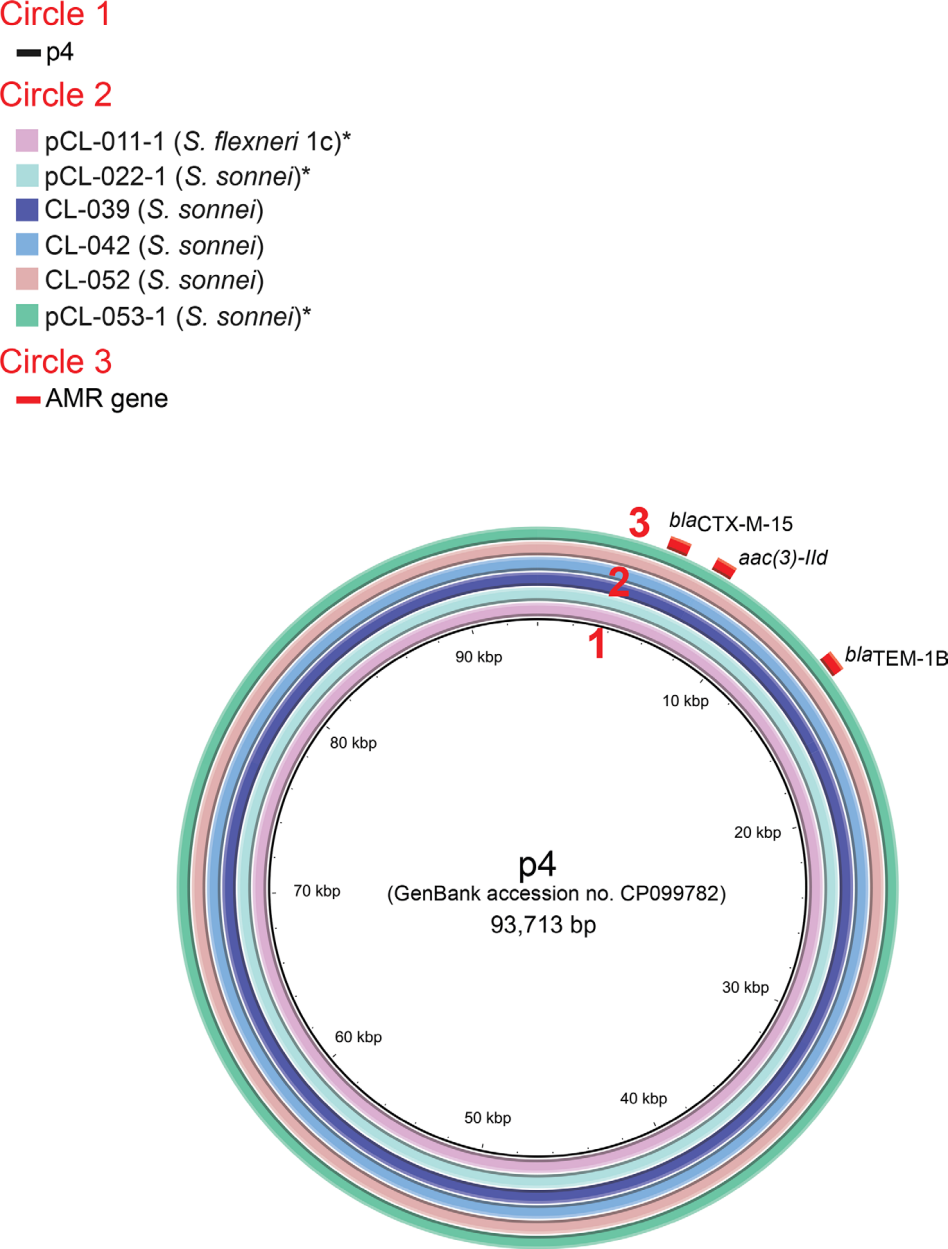
Circular map and comparative analysis of the IncI1 plasmids carrying the ESBL *bla*_CTX-M-15_ gene. Circles from inside to outside indicate: (1) the nucleotide position of p4, a plasmid from *S. sonnei* isolate S14BD05406 collected in Belgium in 2015 [53]; (2) regions of p4 displaying high levels of sequence identity to regions from complete or draft genome assemblies of CL-011 (* S. flexneri* 1c isolate, our study), CL-022 (* S. sonnei* isolate, our study), CL-039 (* S. sonnei* isolate, our study), CL-042 (* S. sonnei* isolate, our study), CL-052 (* S. sonnei* isolate, our study) and CL-053 (* S. sonnei* isolate, our study); and (3) antimicrobial drug resistance (AMR) genes. Asterisks indicate isolates with complete genomes.

However, it proved challenging to obtain a complete sequence for the IncI1 plasmid. We addressed this problem by performing long-read sequencing on five isolates, to explore the genetic characteristics of this plasmid further. The read-mapping and BLASTn (using BRIG) analyses carried on the five circularized plasmids and the six draft assemblies of the 11 ESBL-producing isolates carrying an IncI1 plasmid revealed a high degree of identity and more than 97 % coverage of two different IncI1 plasmids (Figs 3 and 4, Table S1). The first, a plasmid of about 87 kb, carried the *bla*_CTX-M-3_ gene flanked by a remnant *ISEcp1* and the IS*6* family transposase IS*26*.(Table 2). This plasmid was carried by *S. sonnei* and *S. flexneri* (serotypes 2a and 6) isolated from two different hospitals between 2011 and 2016 (Figs 1 and 3, Data S1). It was highly similar (100 % identity, with 98 % coverage) to p7111-69 (GenBank accession no. CP049176), from an *S. sonnei* isolate (7111.69) acquired in Turkey in 2019 (Fig. 3) [43]. The same plasmid was also recently detected in France [9]. The second plasmid, of about 93–96 kb, harboured the *bla*_CTX-M-15_ gene surrounded by IS*26*, together with the *bla*_TEM-1B_ and *aac(3)-IId* genes (Table 2). It was predominantly found in *S. sonnei* isolates but was also carried by one *S. flexneri* serotype 1c isolate (Figs 1 and 4, Data S1). This plasmid was 99 % identical, with >97 % coverage, to p4 (GenBank accession no. CP099782.1) from an *S. sonnei* isolate (S14BD05406) collected in Belgium in 2015 (Fig. 4) [53].

**Table 2.** Complete plasmids obtained by long-read sequencing in our study

Isolate	Year	Serotype	Genotype	AMR plasmid				GenBank accession no.
				Name	Size (kb)	Inc type	AMR genes	
CL-011	2011	* S. flexneri* 1c	PG1a	pCL011-1	96.877	IncI1-I	*bla*_CTX-M-15_ *, bla*_TEM-1B_ *, aac(3)-IId*	OR237793
				pCL011-2	44.261	IncX1	*bla*_TEM-1B_ *, aadA1, dfrA1*	OR237794
CL-046	2014	* S. flexneri* 2a	PG3	pCL046-1	86.105	IncI1-I	*bla*_CTX-M-3_	OR237797
				pCL046-2	6.200	nt	*strA, strB, sul2*	OR237798
CL-022	2012	* S. sonnei *	3.6.1	pCL022-1	93.777	IncI1-I	*bla*_CTX-M-15_ *, bla*_TEM-1B_ *, aac(3)-IId*	OR237795
				pCL022-2	8.401	nt	*strA, strB, sul2, tet(A*)	OR237796
CL-053	2015	* S. sonnei *	3.6.1	pCL053-1	93.766	IncI1-I	*bla*_CTX-M-15_ *, bla*_TEM-1B_ *, aac(3)-IId*	OR237799
				pCL053-2	8.401	nt	*strA, strB, sul2, tet(A*)	OR237800
CL-059	2016	* S. sonnei *	3.6.1	pCL059-1	87.510	IncI1-I	*bla*_CTX-M-3_	OR237801
				pCL059-2	8.401	nt	*strA, strB, sul2, tet(A*)	OR237802

AMR, antimicrobial drug resistance; nt, not typed.

The presence of the same IncI1/IncFIB plasmids in different *Shigella* serogroups (* S. sonnei *, *S. flexneri* and *S. boydii *) isolated from different countries suggests that these plasmids have been successfully transmitted horizontally between different strains and even across continents. This transfer mechanism raises concerns about the dissemination of AMR genes, highlighting the urgent need for enhanced surveillance and the importance of prudent antimicrobial drug use to mitigate the dissemination of antimicrobial drug resistance determinants.

## Conclusion

To our knowledge, this is the first study to provide insight into the serotypes of *Shigella* circulating in Lebanon and the AMR determinants they carry. It revealed a high degree of genetic diversity in the circulating *Shigella* strains, with a marked prevalence of MDR isolates. Our findings also suggest possible interactions between ESBL-producing *Shigella* strains circulating globally and the strains present in Lebanon, although further studies are required to characterize this phenomenon in more detail. Future studies with a larger sample size and enhanced surveillance efforts are warranted to obtain a comprehensive understanding of *Shigella* epidemiology and AMR patterns in Lebanon and beyond.

## Supplementary Data

Supplementary material 1

Supplementary material 2
