# Supplementary material for: Genomic analysis of Shigella isolates from Lebanon reveals marked genetic diversity and antimicrobial resistance
Source: Microb Genom. 2023 Dec 15;9(12):001157. doi: 10.1099/mgen.0.001157 (PMC10763507; doi:10.1099/mgen.0.001157)
Supplement: Supplementary material 1 [file mgen-9-1157-s001.pdf]

Supplementary Table 1: Genomic characterisation and mapping coverage of ESBL-producing *Shigella* isolates based on short reads

					Mapping coverage (%) against plasmids										
Isolate	Serotype	Genotype	Acquired AMR genes	ESBL gene/ plasmid replicon on same contig	pCL011-1 IncI1	pCL011-2 IncX1	pCL046-1 IncI1	pCL046-2 NT	pCL022-1 IncI1	pCL022-2 NT	pCL053-1 IncI1	pCL059-1 IncI1	p38 IncFIB	p4 IncI1	p7111-69 IncI1
CL-011	SF1c	PG1a	*bla*$_{CTX-M-15}$, *bla*$_{TEM-1B}$, *aadA1, aac(3)-IId, dfrA1, tet(B*)	*bla*$_{CTX-M-15}$/IncI1	99.9	100	95.1	41.8	99.9	42.9	100	93.6	4.2	99.9	95.1
CL-014	SS	3.6.1	*bla*$_{CTX-M-3}$, *strA, strB, aac(3)-IId, sul2 dfrA1, tet(A*)	*bla*$_{CTX-M-3}$/IncI1	83.9	13.8	97.5	95.6	86.6	100	86.5	96.	4.8	86.8	97.5
CL-015	SB2		*bla*$_{CTX-M-15}$, *bla*$_{TEM-1B}$, *aac(3)-IId*	*bla*$_{CTX-M-15}$/IncFIB	97.6	5.4	95.8	19.7	100	14.6	100	94.4	96.5	99.9	95.8
CL-022	SS	3.6.1	*bla*$_{CTX-M-15}$, *bla*$_{TEM-1B}$, *strA, strB, aac(3)-IId, sul2, dfrA1, tet(A*)	*bla*$_{CTX-M-15}$/IncI1	97.6	13.6	95.1	96.5	100	100	99.9	93.6	4.7	99.9	95.1
CL-024	SS	3.6.1	*bla*$_{CTX-M-3}$, *strA, strB, sul2, dfrA1, tet(A*)	*bla*$_{CTX-M-3}$/IncI1	85.2	10.9	100	96.6	87.8	100	87.8	98.5	81.6	87.8	100
CL-039	SS	3.6.1	*bla*$_{CTX-M-15}$, *bla*$_{TEM-1B}$, *strA, strB, aac(3)-IId, sul2, dfrA1, tet(A*)	*bla*$_{CTX-M-15}$/IncI1	97.6	13.6	95.2	96.6	100	100	100	93.7	4.7	99.9	95.2
CL-042	SS	3.6.1	*bla*$_{CTX-M-15}$, *bla*$_{TEM-1B}$, *aac(3)-IId, dfrA1*	*bla*$_{CTX-M-15}$/IncI1	97.6	20.7	95.2	9.8	100	7.2	100	93.7	4.9	99.9	95.2
CL-045	SB10		*bla*$_{CTX-M-15}$, *bla*$_{TEM-1B}$, *strA, strB, dfrA14, qnrS1*	*bla*$_{CTX-M-15}$/IncFIB	6.7	13.9	4.2	60.4	6.1	42	6.1	4.1	100	6	4.2
CL-046	SF2a	PG3	*bla*$_{CTX-M-3}$, *bla*$_{OXA-1}$, *strA, strB, aadA1, catA1, sul2, dfrA1, tet(B*)	*bla*$_{CTX-M-3}$/IncI1	85.2	11.7	100	100	87.8	71.5	87.8	98.5	4.6	87.8	100

CL-047	SF6		*bla*$_{\text{CTX-M-3}}$, *aadA1, sul2, dfrA1*	*bla*$_{\text{CTX-M-3}}$ |IncI1	85.2	11.5	99.9	71.5	87.8	50.5	87.8	98.4	4.8	87.7	100
CL-052	SS	3.6.1	*bla*$_{\text{CTX-M-15}}$, *bla*$_{\text{TEM-1B}}$, *strA, strB, aac(3)-IId, sul2, dfrA1, tet(A*)	*bla*$_{\text{CTX-M-15}}$ |IncI1	97.6	14.2	95.1	96.5	99.9	100	100	93.6	4.9	99.9	95.1
CL-053	SS	3.6.1	*bla*$_{\text{CTX-M-15}}$, *bla*$_{\text{TEM-1B}}$, *strA, strB, aac(3)-IId, sul2, dfrA1, tet(A*)	*bla*$_{\text{CTX-M-15}}$ |IncI1	97.6	13.4	95.1	96.5	99.9	100	100	93.7	4.7	99.9	95.1
CL-056	SB20		*bla*$_{\text{CTX-M-15}}$, *aadA1, sul1, dfrA5, tet(A*), *qnrS1*	*bla*$_{\text{CTX-M-15}}$ |IncFIB	85.3	19.9	92.1	24.4	87.2	44.5	87.2	90.6	100	87.2	92.1
CL-059	SS	3.6.1	*bla*$_{\text{CTX-M-3}}$, *strA, strB, sul2, dfrA1, tet(A*)	*bla*$_{\text{CTX-M-3}}$ |IncI1	85.6	11.7	100	96.6	88.2	100	88.2	100	4.8	88.2	100
CL-063	SF6		*bla*$_{\text{CTX-M-15}}$, *aadA1, dfrA1*	*bla*$_{\text{CTX-M-15}}$ |IncFIB	2.9	12	3.6	9.7	3.1	7.1	3	3.5	99.9	3	3.6

SS, *S. sonnei*; SF, *S. flexneri*; SB, *S. boydii*; SD, *S. dysenteriae*; ESBL, extended-spectrum beta-lactamase; AMR, antimicrobial drug resistance; NT, not typed.
